# *OsNAL11* and *OsGASR9* Regulate the Low-Temperature Germination of Rice Seeds by Affecting GA Content

**DOI:** 10.3390/ijms252011291

**Published:** 2024-10-20

**Authors:** Jinzhao Liu, Xi Yuan, Mengqing Tian, Jialing Chen, Chun Chen, Zengtong Luo, Tao Guo, Xing Huo, Wuming Xiao

**Affiliations:** 1 National Engineering Research Center of Plant Space Breeding, South China Agricultural University, Guangzhou 510642, China; ljz999999@stu.scau.edu.cn (J.L.);; 2 Rice Research Institute, Guangdong Academy of Agricultural Sciences, Guangzhou 510640, China

**Keywords:** germinability, GA pathway, low temperature stress, ROS

## Abstract

Low temperatures cause serious threat to rice seed emergence, which has become one of the main limiting factors in the production of direct seeding rice. It is of great importance to study the genes controlling low-temperature tolerance during seed germination and to mine the possible regulatory mechanism for developing new rice varieties with immense low-temperature germination ability. In the current research study, two types of mutants of *nal11* and *gasr9*, derived from the WT (wild type) ZH11, were used for the analysis of low-temperature germinability. The results showed that the *nal11* and *gasr9* mutants displayed no significant difference in germination rate with ZH11 at room temperature, but the mutants showed significantly lower germination rates, germination potential and germination index, and slowed seedling growth in the simulated direct seeding experiments at low temperatures compared to ZH11. Additionally, the activity of POD, SOD, CAT, and anti-superoxide anion radial activity were significantly reduced, but the levels of MDA and H_2_O_2_ were significantly higher in the *nal11* and *gasr9* mutant seeds that were germinated at low temperatures compared to ZH11. Further analysis revealed that the levels of total active GA, especially GA4 and GA7, were significantly lower in the *nal11* and *gasr9* mutants than that in ZH11 during low-temperature germination. Based on qRT-PCR analysis, the expression levels of some GA synthesis-related genes were higher, whereas some were lower in the *nal11* and *gasr9* mutants than those in ZH11, however, the GA metabolism-related genes *OsGA2ox8* and *OsGA2ox10* and the GA signaling negative regulator gene *SLR1* were significantly up-regulated in both *nal11* and *gasr9* mutants at several time points during low-temperature germination. This may explain the lower GA levels in the *nal11* and *gasr9* mutants. Furthermore, the interaction between the OsNAL11 and OsGASR9 proteins was confirmed by Y2H, LUC, and Co-IP assays. This study provides preliminary insights into the regulatory mechanism of the *OsNAL11* and *OsGASR9* genes, which control the low-temperature germination of rice seeds by affecting the GA pathway. Our study will provide the basis for further mining the molecular mechanisms of low-temperature germination in rice and valuable theoretical reference for breeding varieties with strong low-temperature germinability.

## 1. Introduction

Rice (*Oryza sativa* L.) is a widely cultivated crop worldwide and is a staple food for more than half the world’s population. Maintaining the efficient production of rice is of great importance in ensuring food security [1]. With the development of the economy and the increase in labor costs, many countries have changed the traditional rice transplanting cultivation mode and gradually adopted the direct seeding cultivation mode with low production costs and less labor consumption. At present, there are still some problems in the production of direct-seeded rice. In temperate and cold regions, low temperature weather often occurs during the sowing season. In the subtropical regions, sowing in the early cropping season is often followed by low temperatures and cold-water irrigation. Low temperatures will lead to a decrease in rice seed vigor, affect the germination rate and germination speed of rice, cause uneven germination, delayed emergence and even lack of seedlings, and adversely affect the seedling and subsequent growth of rice [2,3]. Therefore, it is of great importance to conduct research on the low-temperature germination (LTG) of rice to develop rice varieties suitable for direct seeding.

The suitable temperature for rice germination ranges from 20 to 33 °C, and most studies use 15 °C as the standard temperature for evaluating the low-temperature germination ability of rice seeds [4]. Although a large number of quantitative trait loci (QTLs) associated with LTG have been reported, only a few genes controlling LTG have been cloned so far. *qLTG-3-1* is the first cloned gene related to LTG. The *qLTG-3-1* gene, which encodes a HyP/GRP protein containing 184 amino acids, can regulate the vacuolization of the tissues around the embryo and reduce the mechanical resistance to seed germination, thereby improving the low-temperature germination ability of rice seeds [5,6]. *OsSAP16*, a gene encoding a stress protein with two C2H2-AN1 zinc finger domains, was found to control LTG in rice seeds. The loss-of-function mutants of the *OsSAP16* gene had very low temperature germination rates, and its overexpression lines had a significantly better low-temperature germination ability than the wild type [7]. A 4 bp InDel in the *GF14h* gene is reported to alter the germination rate of rice seeds at an optimal temperature by genome-wide association analysis. The GF14h protein, the bZIP transcription factor OREB1, and the florigen-like protein MFT2 form a transcriptional regulatory module to control germination rate by regulating abscisic acid (ABA)-responsive genes. The loss-of-function allele of *GF14h* increased ABA signaling and decreased germination rate. This allele is present in rice varieties in the northern region and in modern varieties in Japan and China, suggesting that it contributes to the geographical adaptability of rice [8,9]. *OsUBC12*, encoding an E2 ubiquitin-conjugating enzyme, increases low-temperature germinability in japonica, by negatively regulating ABA signaling. Its regulation of seed germination and ABA signaling depends mainly on a conserved active site required for ubiquitin-conjugating enzyme activity [10].

Gibberellins (GAs) are a very important class of plant hormones that play a major role in seed germination [11]. The *OsPK5* gene, encoding pyruvate kinase, regulates rice seed germination by affecting GA levels through the glycolysis pathway [12]. Exogenous GA3 regulates the balance of endogenous hormones, enhances the activities of key enzymes, and reduces the accumulation of active oxygen, thereby accelerating the metabolism and conversion of materials and improving the low-temperature tolerance of cotton seeds during the germination stage [13]. Co-expression of OsDOR1 with OsGID1 in rice protoplasts attenuated the GA-dependent degradation of OsSLR1, the key repressor of GA signaling, and the endogenous OsSLR1 protein level in the *dor1* mutant seeds is significantly lower than that of the wild type. The *dor1* mutant featured a hypersensitive GA-response of α-Amylase gene expression during seed germination [14]. Exogenous bioactive GA3 increased starch hydrolysis and sugar consumption to boost rice seed germination [15].

Our previous study found that the *OsNAL11* gene, which encodes a DnaJ domain-containing heat shock protein (HSP), can regulate rice plant type by affecting the GA content of rice plants [16]. Given that GA is also an important factor affecting seed germination, we, therefore, speculate that the *OsNAL11* gene may also play a role in rice seed germination.

In this study, the mutants derived from the knockout of the *OsNAL11* gene were found to have no significant difference in germination ability from the wild type (WT) at room temperature, but their germination ability at low temperatures was significantly worse than that of the WT. Further analysis revealed that the OsNAL11 protein interacts with the OsGASR9 protein. *OsGASR9*, a member of the gibberellic acid-stimulated transcript (GAST) family genes, has been reported to positively regulate grain size and yield in rice and is involved in the GA pathway [17], but its role in regulating seed germination at low temperatures has not been elucidated. It is speculated that the effects of *OsNAL11* and *OsGASR9* genes on seed germination at low temperatures may be related to GA. Therefore, this study focused on the GA pathway and physiological changes to reveal the possible regulation mechanism involved in rice seed germination under low-temperature stress. Thus, this study can enrich the understanding of the regulation mechanism of rice seed germination at low temperatures, and provide a favorable reference and basis for breeding cold-tolerant varieties at the germination stage, laying the foundation for the development of direct-seeded rice.

## 2. Results

### 2.1. OsNAL11 Gene Affected Seed Germination and Seedling Growth Under Low-Temperature Conditions

It was observed that ZH11 and the *nal11-1* and *nal11-2* mutants started to germinate on day one at room temperature, and their seeds germinated completely after three days. There were no significant differences in germination rates between ZH11 and the *nal11-1* and *nal11-2* mutants. In addition, the germination potential and T50 (time required for 50% seed germination) of both mutants did not reach a significant level compared to that of ZH11 (Figure S1). Thus, knocking out the *OsNAL11* gene had a marginal effect on seed germination at room temperature.

**Figure 1:**
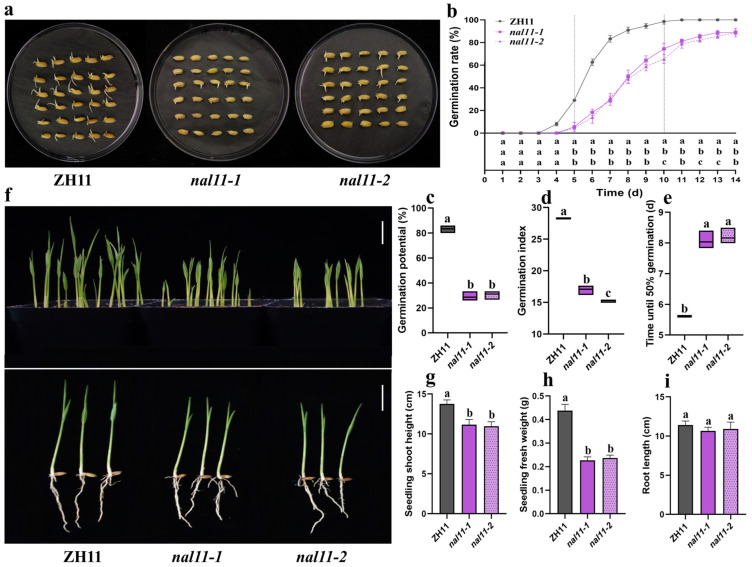
Seed germination and seedling growth of ZH11, *nal11-1*, and *nal11-2* mutants at low temperatures. (**a**) Seed germinating status of ZH11, *nal11-1*, and *nal11-2* mutants at 15 ± 1 °C for 12 days. (**b**) Germination rates of ZH11 and two mutants at 15 ± 1 °C. (**c**–**e**) Germination potential (seven days), germination index, and T50 (time required for seeds to reach 50% germination) of ZH11 and two mutants at 15 ± 1 °C, respectively. (**f**) The seedlings of ZH11, *nal11-1*, and *nal11-2* mutants on day nine of simulated direct seeding at 18 ± 1 °C, bar=2.0 cm. (**g**) Seedling shoot height of ZH11, *nal11-1*, and *nal11-2* mutants after 14 days of direct seeding treatment at 18 ± 1 °C. Sample size was 10. (**h**) Seedling fresh weight of ZH11, *nal11-1*, and *nal11-2* mutants after 14 days of direct seeding treatment at 18 ± 1 °C. 10 seedlings were randomly selected each time, and three biological replicates were performed. (**i**) Root length of ZH11, *nal11-1*, and *nal11-2* mutants after 14 days of direct seeding treatment at 18 ± 1 °C. Sample size was 10. All values are presented as the mean ± SD. *n* = 3. The above data were analyzed for significance of differences by one-way ANOVA for each group of data using the LSD method. The letters “a, b, c” indicate *p* < 0.05, in Figure (**b**), the letters in the figure stand for ZH11, *nal11-1*, and *nal11-2* from top to bottom.

At a low temperature of 15 ± 1 °C, the overall germination status of the *nal11-1* and *nal11-2* mutant seeds was not as good as that of the wild type ZH11 (Figure 1a). ZH11 began to germinate on day four, while the two mutants started germination on day five. The germination rates of ZH11 and the *nal11-1* and *nal11-2* mutants showed significant differences on day five. The germination rate of ZH11 was close to 100% on day 10, while a few seeds of the *nal11-1* and *nal11-2* had still failed to germinate on day 14. In general, the germination rates of the *nal11-1* and *nal11-2* mutants were lower than those of ZH11 on days 5 to 14 (Figure 1b). The germination potential and germination index of the two mutants were also significantly lower than those of ZH11. As for the T50, the mutants were about two days longer than that of ZH11 (Figure 1c–e).

The results of simulated direct seeding experiments at low temperatures showed that the *nal11-1* and *nal11-2* mutants exhibited delayed germination and slowed seedling growth compared to ZH11 (Figure 1f). After 14 days, the seedling shoot height and seedling fresh weight of ZH11 were significantly higher than those of *nal11-1* and *nal11-2*, while the difference in seedling root length was not significant (Figure 1g–i). Thus, the *nal11-1* and *nal11-2* mutants showed poor germination and seedling growth at low temperatures.

### 2.2. Physiological Indices of Low-Temperature Germinated Seeds Were Altered in nal11 Mutants

**Figure 2:**
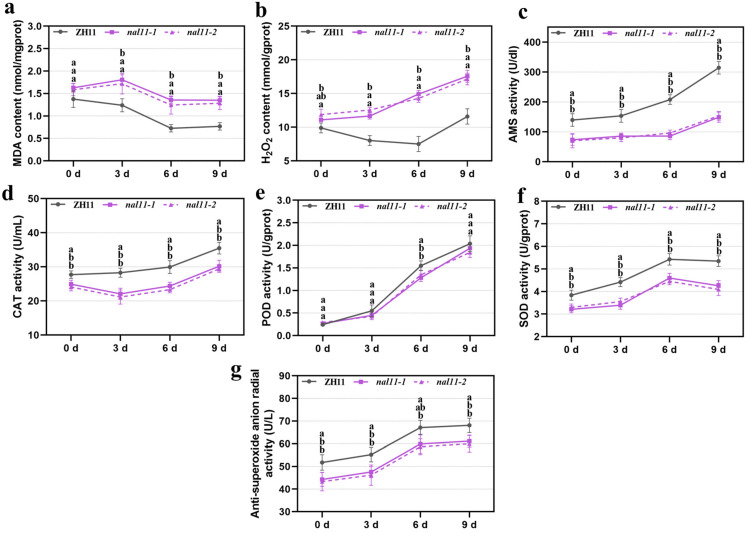
Comparison of physiological indices between ZH11, *nal11-1*, and *nal11-2* mutant seeds germinated at low temperatures. (**a**–**g**) means MDA, H_2_O_2_, AMS, CAT, POD, SOD, and anti-superoxide anion activity, respectively. All values are presented as the mean ± SD. *n* = 3. Data from each group were analyzed using one-way ANOVA, and the significance of differences was analyzed using the LSD method. The letters “a, b” indicate *p* < 0.05, the letters in the figure stand for ZH11, *nal11-1*, and *nal11-2* from top to bottom, and three biological replicates were performed.

The physiological status in seeds plays an important role in low-temperature germination, so it is necessary to determine some key physiological indices between ZH11 and the *nal11-1* and *nal11-2* mutants, such as α-AMS (α-Amylase) level, MDA (malondialdehyde) level, H_2_O_2_ level, anti-superoxide anion radial activity, POD (peroxidase) activity, SOD (superoxide dismutase) activity, and CAT (catalase) activity. The results showed that the mutants exhibited a significantly lower α-AMS level, SOD activity, and anti-superoxide anion activity than those of ZH11 during low-temperature germination on days zero, three, six, and nine. With the exception of day zero, the CAT activity of the mutants was also significantly lower than that of ZH11 on days three, six, and nine. Regarding the POD activity, there was no significant difference between the two mutants and ZH11 at different time points. In contrast, the levels of MDA and H_2_O_2_ of the mutants were significantly higher than those of ZH11 on days three, six, and nine during low-temperature germination (Figure 2). Thus, the active oxygen scavenging ability of the mutants was destroyed to some extent during low-temperature germination, resulting in the accumulation of H_2_O_2_ and MDA, and the overall physiological status was not as good as that of ZH11.

### 2.3. OsNAL11 Interacts with OsGASR9

**Figure 3:**
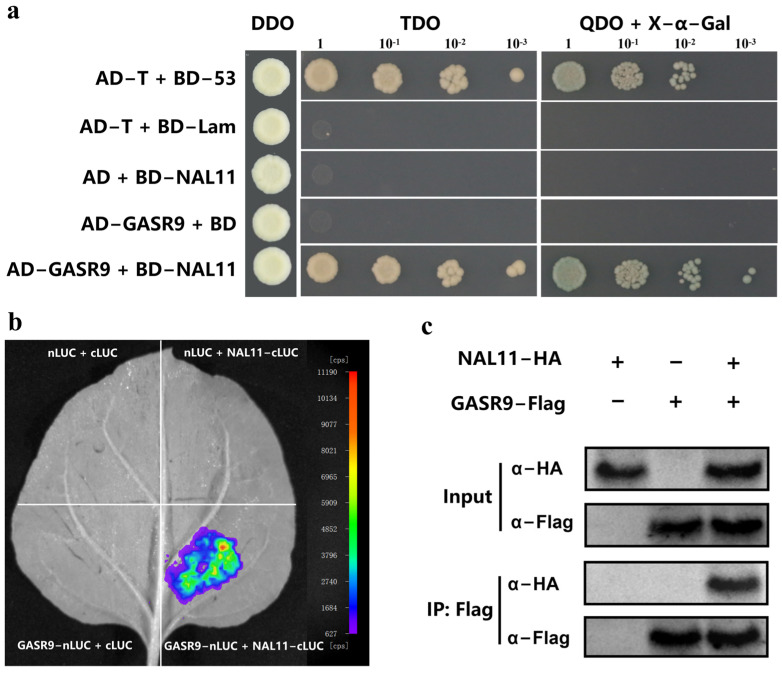
OsNAL11 interacts with OsGASR9 in vitro and in vivo. (**a**) Measurement of the interaction between OsNAL11 and OsGASR9 by the yeast two-hybrid (Y2H) assay. Transformed yeasts were spotted on SD-Trp-Leu-His (TDO) or SD-Trp-Leu-His-Ade (QDO) with X-α-Gal medium in 10-, 100-, 1000-fold dilution. Empty vectors served as the controls. (**b**) Firefly luciferase (LUC) complementation imaging assay. NAL11-cLUC and GASR9-nLUC with the control vector were co-infiltrated into *N. benthamiana* leaves. LUC images were captured using a cooled charge-coupled device (CCD) imaging apparatus. (**c**) The Co-IP assay illustrating the interaction between OsNAL11 and OsGASR9. GASR9-Flag coupled magnetic beads were used to precipitate NAL11-HA proteins from the rice protoplasts. The experiment was repeated three times with similar results.

To elucidate the underlying mechanism by which OsNAL11 affects seed germination, we performed a yeast two-hybrid (Y2H) assay using *pGBKT7-NAL11* as a bait to screen a cDNA library, which was constructed in the prey vector *pGADT7* with cDNAs from germinated seeds and buds of ZH11. OsGASR9 was identified as a candidate interactor (Figure 3a). The LUC assay also verified the interaction of OsNAL11 with OsGASR9 in tobacco leaves. The results showed that when the tobacco leaves were co-transformed with *pCAMBIA1300-NAL11-cLUC* and *pCAMBIA1300-GASR9-nLUC* together, rather than various negative controls, the clear LUC signal was found (Figure 3b). Furthermore, the Co-IP assay showed that the NAL11-HA was detected by an anti-HA antibody in the immunoprecipitated proteins with an anti-Flag antibody only when both the *pRTVcHA-NAL11* and *pRTVcFlag-GASR9* plasmids were co-transformed into rice protoplasts, but not when *pRTVcHA-NAL11* or *pRTVcFlag-GASR9* alone together with the corresponding empty vector plasmid were transformed.(Figure 3c). These results demonstrated that OsNAL11 interacts with OsGASR9 in vitro and in vivo. *OsGASR9*, as a member of the gibberellic acid-stimulated transcript (GAST) family genes [17], has not been elucidated in its role in regulating seed germination at low temperatures.

### 2.4. The gasr9 Mutants Showed Reduced Seed Germination and Seedling Growth Under Low-Temperature Conditions

In this study, the *gasr9-1* and *gasr9-2* mutants were constructed by knocking out the *OsGASR9* gene in the ZH11 background to investigate whether it also affects the low-temperature germination of rice seeds. The results showed that at room temperature, the germination rates of ZH11 and the *gasr9-1* and *gasr9-2* mutants were close to 100.0% after 2.5 days, and there was no significant difference in germination rate, germination potential and T50 between them (Figure S2).

**Figure 4:**
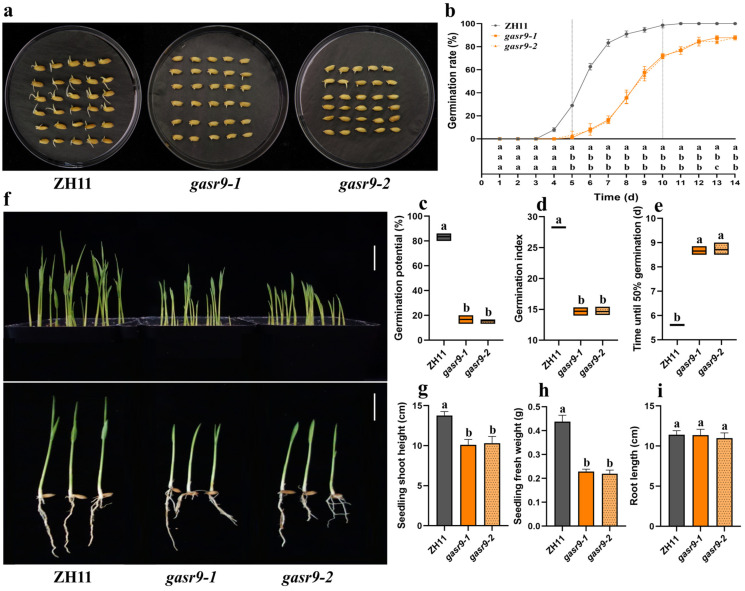
Seed germination and seedling growth of ZH11, *gasr9-1*, and *gasr9-2* mutants at low temperatures. (**a**) Seed germinating status of ZH11, *gasr9-1*, and *gasr9-2* mutants at 15 ± 1 °C for 12 days. (**b**) Germination rates of ZH11 and two mutants at 15 ± 1 °C. (**c**–**e**) Germination potential (seven days), germination index, and T50 (time required for seeds to reach 50% germination) of ZH11 and two mutants at 15 ± 1 °C, respectively. (**f**) The seedlings of ZH11, *gasr9-1*, and *gasr9-2* mutants on day nine of simulated direct seeding at 18 ± 1 °C, bar = 2.0 cm. (**g**) Seedling shoot height of ZH11, *gasr9-1*, and *gasr9-2* mutants after 14 days of direct seeding treatment at 18 ± 1 °C. Sample size was 10. (**h**) Seedling fresh weight of ZH11, *gasr9-1*, and *gasr9-2* mutants after 14 days of direct seeding treatment at 18 ± 1 °C. 10 seedlings were randomly selected each time, and three biological replicates were performed. (**i**) Root length of ZH11, *gasr9-1*, and *gasr9-2* mutants after 14 days of direct seeding treatment at 18 ± 1 °C. Sample size was 10. All values are presented as the mean ± SD. *n* = 3. The above data were analyzed for significance of differences by one-way ANOVA for each group of data using the LSD method. The letters “a, b” indicate *p* < 0.05, in Figure (**b**), the letters in the figure stand for ZH11, *gasr9-1*, and *gasr9-2* from top to bottom.

At a low temperature of 15 ± 1 °C, *gasr9-1* and *gasr9-2* mutant seeds showed poor germination status compared to ZH11 (Figure 4a). ZH11 began to germinate on day four, and its germination rate was close to 100% on day 10. On the other hand, the *gasr9-1* and *gasr9-2* mutants started to germinate on day five, and some seeds of the two mutants still failed to germinate on day 14. Thus, the germination rates of the *gasr9-1* and *gasr9-2* mutants were lower than those of ZH11 on days 5 to 14 (Figure 4b). The *gasr9-1* and *gasr9-2* mutants also showed significantly lower germination potential and germination index compared to ZH11 (Figure 4c,d). The T50 of the two mutants was about two and a half days longer than that of ZH11 (Figure 4e).

Similarly, in the simulated direct seeding experiments at low temperatures, the *gasr9-1* and *gasr9-2* mutants showed delayed germination and slowed seedling growth compared to ZH11 (Figure 4f). The two mutants also showed significantly lower seedling shoot height and seedling fresh weight at day 14 than those of ZH11, with no significant difference in seedling root length (Figure 4g–i). Thus, knocking out the *OsGASR9* gene also resulted in the reduced germination and seedling growth of rice seeds under low-temperature conditions.

**Figure 5:**
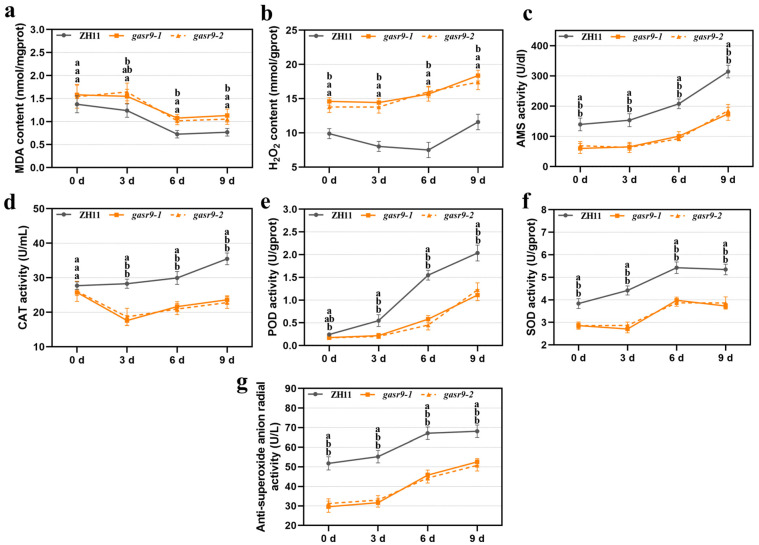
Comparison of physiological indices between ZH11, *gasr9-1*, and *gasr9-2* mutant seeds germinated at low temperatures. (**a**–**g**) means MDA, H_2_O_2_, AMS, CAT, POD, SOD, and anti-superoxide anion activity, respectively. All values are presented as the mean ± SD. *n* = 3. Data from each group were analyzed using one-way ANOVA, and the significance of differences was analyzed using the LSD method. The letters “a, b” indicate *p* < 0.05, the letters in the figure stand for ZH11, *gasr9-1*, and *gasr9-2* from top to bottom, and three biological replicates were performed.

The results of the physiological index assay showed that the H_2_O_2_ level in *gasr9-1* and *gasr9-2* mutants was significantly higher than that of ZH11 both before and during low-temperature germination. The MDA level of *gasr9-1* and *gasr9-2* mutants was significantly higher than that of ZH11 on days six and nine of low-temperature germination. However, the α-AMS level, anti-superoxide anion activity, and SOD activity of the two mutants were significantly lower than those of ZH11 at different time points before and after low-temperature germination. The CAT and POD activities of the two mutants were also significantly lower than those of ZH11 on days three, six, and nine (Figure 5). It is clear that knocking out the *OsGASR9* gene also affects the physiological indices of low-temperature germinated seeds.

### 2.5. The nal11 and gasr9 Mutants Reduced the GA Levels in Seeds Germinated at Low Temperatures

**Figure 6:**
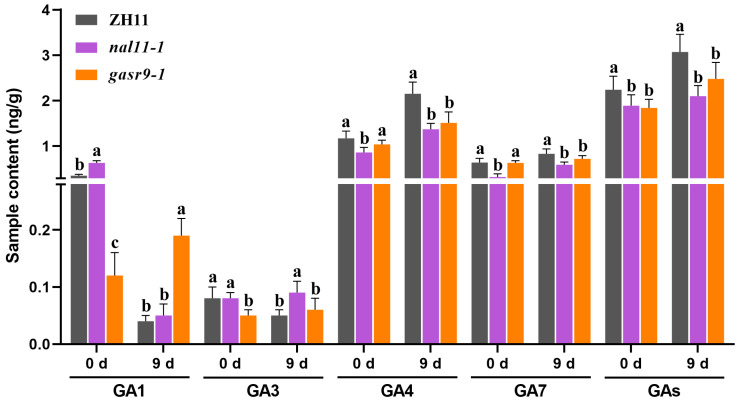
Endogenous bioactive GA levels of ZH11 and the *nal11-1* and *gasr9-1* mutants during low-temperature germination. The values are presented as the mean ± SD. *n* = 3. Data from each group were analyzed using one-way ANOVA, and the significance of differences was analyzed using the LSD method. The letters “a, b, c” indicate *p* < 0.05.

In this study, the levels of the endogenous active GA components GA1, GA3, GA4, and GA7 were determined in the seeds of ZH11, *nal11-1*, and *gasr9-1* germinated at low temperatures on days zero and nine. The results showed that before germination (zero days), the GA1 level of *nal11-1* was significantly higher than that of ZH11, and the GA3 level was not significantly different from that of ZH11, but the levels of GA4 and GA7 were significantly lower than those of ZH11 (Figure 6). On day nine, the GA1 level of *nal11-1* decreased and was close to that of ZH11, while the GA3 level of *nal11-1* was almost the same as on day zero, but significantly higher than that of ZH11. Although the GA4 level of both the ZH11 and *nal11-1* increased, the increase in *nal11-1* was less, resulting in a significantly lower GA4 level than that of ZH11. The GA7 level of *nal11-1* was also significantly lower than that of ZH11. In the *gasr9-1* mutant, the levels of GA1, GA3, and GA4 were lower than in ZH11 before germination. On day nine of low-temperature germination, the levels of GA1 and GA3 decreased, but the levels of GA4 and GA7 increased significantly in ZH11, whereas the levels of GA1, GA3, and GA4 increased in *gasr9-1*. Although the level of GA1 was significantly higher than that of ZH11, the levels of GA4 and GA7 were still significantly lower than that of ZH11. In general, the level of total active GAs (the sum of GA1, GA3, GA4, and GA7) in the *nal11-1* and *gasr9-1* mutants was significantly lower than that in ZH11 both before germination (day zero) and on day nine of low-temperature germination (Figure 6). Since the levels of GA4 and GA7 are much higher than those of GA1 and GA3, it is speculated that the decrease in the levels of GA4 and GA7 in the *nal11* and *gasr9* mutant seeds may be one of the important factors affecting the low-temperature germination ability.

### 2.6. Both the OsNAL11 and OsGASR9 Genes Were Involved in Regulating the Expression of GA Pathway-Related Genes During Low-Temperature Germination

The above results indicated that the *nal11* and *gasr9* mutants significantly reduced the levels of bioactive GA in seeds during low-temperature germination. Accordingly, we used qRT-PCR to investigate the expression levels of genes involved in GA synthesis, metabolism and signal transduction in ZH11, *nal11*, and *gasr9* mutant seeds during low-temperature germination.

**Figure 7:**
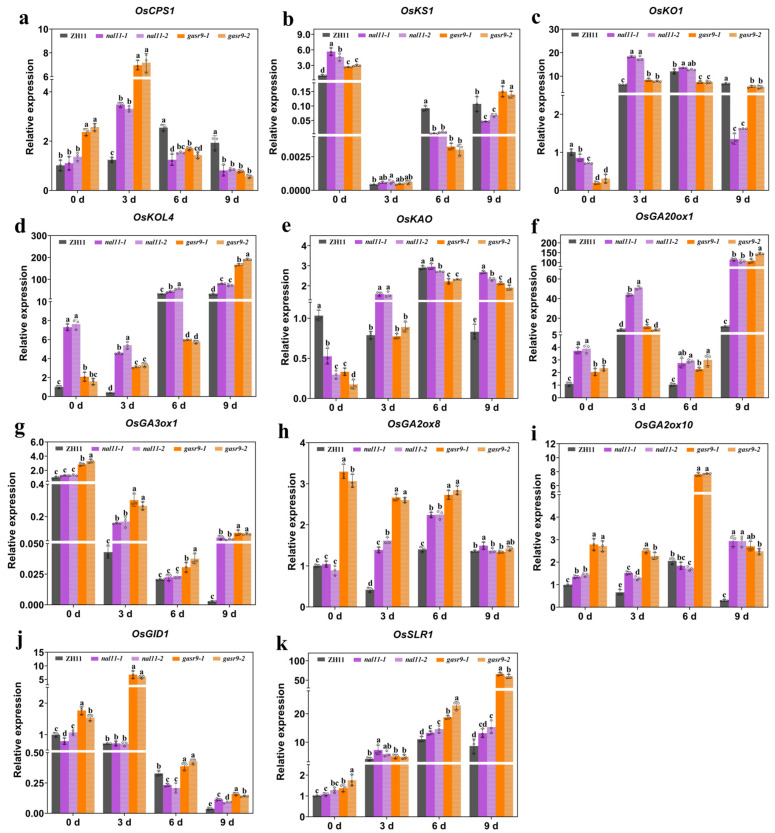
Expression of GA pathway-related genes in ZH11 and the *nal11* and *gasr9* mutants during low-temperature germination. Subfigures (**a**–**g**) show the expression changes of genes involved in gibberellin biosynthesis in ZH11 and the *nal11* and *gasr9* mutants under low temperature; (**h**,**i**) represent the expression changes of genes involved in gibberellin biometabolism under low temperature; (**j**,**k**) represent the expression changes of genes involved in the gibberellin signal transduction pathway under low temperature. All values are presented as the mean ± SD. *n* = 3. The expression at zero days in the ZH11 was used as the control and the relative expression was calculated for three biological replicates. The data of the above groups were analyzed by one-way ANOVA, and the significance of the differences was analyzed by the LSD method, and the letters “a, b, c, d and e” indicate *p* < 0.05.

The results showed that the expression levels of *OsKS1*, *OsKOL4*, and *OsGA20ox1* genes in the GA synthesis pathway in the *nal11* mutants and the expression levels of *OsCPS1*, *OsGA20ox1*, and *OsGA3ox1* genes in the *gasr9* mutants were significantly higher than those in ZH11 before germination (zero hours), but the expression levels of the *OsKO1* and *OsKAO* genes were significantly lower in the two type mutants than in ZH11. The expression level of the *OsCPS1* gene reached the highest value in ZH11 on day six, and the highest value in the *nal11* and *gasr9* mutants appeared on day three, which was significantly higher than that of ZH11, but significantly lower than that of ZH11 on days six and nine (Figure 7a). In general, the *OsKS1* gene showed a low expression level on days three, six, and nine during low-temperature germination, which was extremely low on day three, and its expression level in the *nal11* and *gasr9* mutants was significantly lower than that in ZH11 on day six (Figure 7b). Compared with that before germination (zero days), the expression level of the *OsKO1* gene increased on days three, six, and nine. The expression level of the *OsKO1* gene in the *nal11* and *gasr9* mutants was significantly higher than that in ZH11 on day three, but significantly lower than that in ZH11 on day nine (Figure 7c). The expression level of the *OsKAO* gene was relatively high on day six, and its expression levels on days three and nine were significantly higher in the *nal11* mutants than in ZH11 (Figure 7e). In the *gasr9* mutants, its expression level was significantly lower than that of ZH11 on day six, but significantly higher than that of ZH11 on day nine. The expression level of the *OsGA20ox1* gene in the *nal11* mutants was significantly higher than that in ZH11 on day three during low-temperature germination. Its expression level in the *nal11* and *gasr9* mutants was significantly higher than that in ZH11 on days six and nine (Figure 7f). Collectively, its expression level in the *nal11* mutants was significantly higher than that in ZH11 throughout low-temperature germination. The *OsGA3ox1* gene showed a very low expression level during low-temperature germination compared to that before germination (zero days) (Figure 7g). Thus, in the *nal11* and *gasr9* mutants, the expression levels of some GA synthesis-related genes were higher than in ZH11, and some were lower than in ZH11, and the expression levels at different time points were also different.

Before germination (zero days), the expression levels of the GA metabolism-related genes *OsGA2ox8* and *OsGA2ox10* were close to each other in ZH11 and the *nal11* mutants, but significantly higher in the *gasr9* mutants than in ZH11. The expression of the *OsGA2ox8* gene was significantly higher in the two type mutants than in ZH11 on days three and six (Figure 7h). The expression of the *OsGA2ox10* gene was also significantly higher in the two type mutants than in ZH11 on days three and nine, and also significantly higher in the *gasr9* mutants than in ZH11 on day six (Figure 7i). In general, the expression levels of the *OsGA2ox8* and *OsGA2ox10* genes were significantly higher than those of ZH11 in both *nal11* and *gasr9* mutants at several time points. In the GA signaling pathway, the expression level of the GA receptor gene *GID1* was significantly decreased on days six and nine (Figure 7j). Its expression level was significantly higher than that of ZH11 in the *gasr9* mutants on days zero, three, six and nine, but there was no significant difference between the *nal11* mutants and ZH11 on days zero and three. The expression level of the rice DELLA protein gene *SLR1* showed a gradual increase, and its expression level was significantly higher than that of ZH11 in both the *nal11* and *gasr9* mutants on days six and nine during low-temperature germination (Figure 7k), suggesting that more DELLA proteins may accumulate in the *nal11* and *gasr9* mutants to block the downward transmission of GA signals.

## 3. Discussion

Under normal conditions, the production and scavenging of ROS (reactive oxygen species) in rice is maintained at homeostasis. When the plant is stressed by adversity, ROS levels in the plant increase dramatically, and excessive ROS can cause cell damage [18]. Cold stress increased the levels of ROS and MDA, causing electrolyte leakage in the cells, deteriorating the fluidity of cell membranes, disrupting the stability of the protein complexes, and damaging the internal structure of the seeds [19]. MDA reflects the degree of membrane lipid peroxidation in plant cells, and its levels are closely related to plant senescence and stress damage. Antioxidant enzyme systems such as SOD, CAT, and POD are the main factors in ROS scavenging in plants. The activity of SOD, CAT, and POD is inhibited in seeds during germination under stress, but the ROS-scavenging enzymes tend to remain more active in varieties with a strong low-temperature germination ability [20]. It was found that the direct-seeded rice varieties with low-temperature tolerance during germination had higher soluble sugar content and SOD activity [18]. In this study, the SOD and CAT activities of *nal11* and *gasr9* mutant seeds were significantly lower than those of ZH11 during low-temperature germination, and the POD activity of *nal11* mutants was also significantly lower than that of ZH11. The results showed that the antioxidant enzyme systems of the *nal11* and *gasr9* mutants were affected to some extent, resulting in reduced ROS-scavenging capacity. As a result, the H_2_O_2_ and MDA levels of *nal11* and *gasr9* mutant seeds were significantly higher than those of the WT during low-temperature germination, implying that the low temperature caused more severe damage to *nal11* and *gasr9* mutants than to the WT, which to some extent accounts for the reduced low-temperature germination ability of *nal11* and *gasr9* mutants. Studies have shown that the homologous genes of *OsNAL11* and *OsGASR9* are closely related to reactive oxygen species scavenging. OsHSP60-3B interacts with FLO6 to regulate starch granule biogenesis in rice pollen and attenuates ROS levels in anthers to ensure normal male gametophyte development in rice [21]. The *cpHSC70-1* is induced and functions positively in a plant’s response to osmotic stress by promoting the expression of the stress-responsive genes and reducing ROS accumulation [22]. Hydrogen peroxide was accumulated at high levels in heat stress-treated *GASA5*-overexpressing plants [23]. Other reports support that *AtGASA14* is regulated by abiotic stresses [24]. It was reported that all *TdGASA* genes seem to be responsive to salt and osmotic stress, and the overexpression of *TdGASA* genes in yeast showed better growth compared to non-transgenic control yeast under salt, osmotic, LiCl, H_2_O_2_, and heat stress [25]. Further research is needed to understand how the *NAL11* and *GASR9* genes are involved in ROS scavenging in rice seeds during low-temperature germination.

The α-Amylase plays a critical role in seed germination by degrading starch in the endosperm to provide energy [26]. Low-temperature treatments tend to inhibit carbohydrate degradation and reduce total organic acid content and α-Amylase activity in rice seeds [27]. Low-temperature stress induces the expression of *OsMYB30*, which in turn activates the expression of *OsTPP1* and increases trehalose content, thereby inhibiting α-Amylase activity and seed germination [28]. In the primed rice seeds, GA induces the expression of the α-Amylase genes, enhances starch degradation in seeds under cold stress, and improves seed germination at low temperatures [29]. In this study, we found that the α-Amylase activities of both *nal11* and *gasr9* mutant seeds were significantly lower than that of ZH11, which might be related to the reduced GA levels of the mutants or to the involvement of *OsNAL11* and *OsGASR9* genes in the regulation of α-Amylase activity. In conclusion, the decrease of α-Amylase activity also explains the reduced low-temperature germination ability of the mutants.

Plant hormones play a key role in seeds, mainly abscisic acid (ABA) and gibberellin (GA), are the major endogenous factors that act antagonistically in the control of seed dormancy and germination; ABA positively regulates the induction and maintenance of dormancy, while GA enhances germination [30,31]. Under low-temperature conditions, the level of bioactive GA in rice seeds is reduced and GA signaling is inhibited, which reduces starch hydrolysis and sugar consumption in seeds, resulting in a decrease in seed germination rate [32]. *OsGPq3* controls rice viviparous germination under low-temperature conditions through participation in the GA and ABA signaling pathways [33]. Cucumber *qLTG1.1* was identified as the *CsGAI* gene, which encodes a DELLA family protein involved in regulating the expression of *CsGA2ox* and *CsGA3ox* genes to regulate the germination of cucumber seeds at low temperatures [34]. Considering that the *OsGASR9* gene encodes a GA-regulated protein, it belongs to the GASA superfamily. It is speculated that this gene may regulate the low-temperature germination of rice seeds by participating in the GA pathway. Since the OsNAL11 protein interacts with OsGASR9, it is speculated that the *OsNAL11* gene is also involved in the GA pathway. Therefore, this study focused on the active GA levels of the low-temperature germinated seeds of *nal11* and *gasr9* mutants. The results showed that both *nal11* and *gasr9* mutants had significantly lower levels of GA4, GA7, and active GA than ZH11 on day nine during low-temperature germination, confirming our speculation that both the *OsNAL11* and *OsGASR9* genes are involved in the GA pathway.

A previous study showed that the *OsNAL11* gene, which encodes an HSP40 protein containing a DnaJ domain, regulates rice plant architecture by modulating GA homeostasis [16]. HSPs include five subfamilies, namely the sHSPs, HSP60, HSP70, HSP90, and HSP100 families. The molecular weight of sHSPs (small heat shock proteins) is about 12~40 kDa, mainly including HSP20 and HSP40 [35]. Studies have shown that HSPs are involved in many biological processes such as seed germination, plant growth and development, and abiotic stress response. HSP70-16 interacts with VDAC3 and facilitates the opening of the VDAC3 ion channel, which influences ABA efflux from endosperm to embryo, thus, negatively regulating seed germination in *Arabidopsis* under cold stress [36]. Some HSPs can act as sensors of cellular stress, directly sense reactive oxygen species (ROS), and regulate the expression of oxidative stress response genes in the process of oxidative stress [37]. High temperatures, low temperatures, drought, strong light, and other stresses can induce the expression of J-type HSP proteins [38]. The members of the sHSP family genes, such as *sHsp17.7-CI* and *sHsp21-P*, are involved in the low-temperature response of tomatoes during cold storage. The levels of sHSPs were significantly lower in chilling-sensitive varieties than in chilling-tolerant varieties [39]. An ER-localized small heat shock protein, WAP20, accumulates in the bark tissue of the mulberry tree (*Morus bombycis* Koidz.) during seasonal cold acclimation, which enhances the tolerance to cold stress [40]. As an HSP gene, *OsNAL11* is considered to be involved in the tolerance of plants to stress. Sure enough, the reduced low-temperature germination ability of *nal11* mutants in this study confirms our speculation. OsGASR9, located in the nucleus and cytoplasm, belongs to the GAST (gibberellic acid-stimulated transcript) family [17]. The GAST gene family is very widely distributed and numerous, and is extensively involved in plant growth and development. *GASA5* is a negative regulator of GA-induced flowering and stem growth in *Arabidopsis* [41]. Although overexpression of both *GASA4* and *GASA5* genes in *Arabidopsis* increased redox activity to some extent and could inhibit the accumulation of ROS [42], in contrast to GASA4, which promotes seed germination [43], GASA5 suppresses GA-induced germination. *AtGASA6* regulates seed germination by promoting hypocotyl cell elongation, resulting in increased hypocotyl length [44]. Overexpression of *OsGASR1* in rice improves the salt tolerance of transgenic plants by reducing H_2_O_2_ levels [45]. *PpyGAST1*, a member of the GAST gene family in pears, was rapidly up-regulated during bud dormancy and was involved in up-regulating *PpyGA20ox2* and increasing active GA levels. Overexpression of *PpyGAST1* in *Arabidopsis* resulted in higher expression levels of *AtGA20ox2* and *AtGA3ox1* [46]. *OsGASR9* has been reported to regulate plant height, panicle type, and grain size by influencing the growth of cells [17]. Though the role of this gene in regulating seed germination at low temperatures is reported here for the first time, the regulatory mechanism needs to be further investigated.

In this study, we found that both *OsNAL11* and *OsGASR9* genes regulate seed germination at low temperatures, probably through the GA pathway. Further analysis revealed that although some genes in the GA synthesis pathway were more highly expressed in *nal11* and *gasr9* mutants than in ZH11 at one or several time points, the key genes responsible for GA metabolism, *GA2ox8* and *GA2ox10*, were also significantly up-regulated in *nal11* and *gasr9* mutants than in ZH11 at multiple time points. The results provide a good explanation for the lower levels of active GA in *nal11* and *gasr9* mutants than in ZH11. In addition, the expression levels of *SLR1*, a key gene in the GA signaling pathway, were significantly higher in both *nal11* and *gasr9* mutants than in ZH11 at several time points. *SLR1* encodes a DELLA protein, which is a negative regulator of GA signaling and acts as an inhibitor of seed germination and plant growth. It has been reported that a low temperature leads to an increase in the expression of the *SLR1* gene by reducing GA levels [47]. These results further indicate that the *OsNAL11* and *OsGASR9* genes affect GA levels by influencing the expression of GA pathway-related genes, thereby regulating the germination of rice seeds under low-temperature stress.

HSPs have important biological functions because they can act as molecular chaperones involved in intracellular protein folding, assembly, transport, etc., and can defend against adverse environmental conditions [48]. The involvement of GASA genes in plant growth and stress responses was mediated by DELLA and heat-shock proteins [49]. Considering the interactions between the OsNAL11 and OsGASR9 proteins, it is speculated that OsNAL11 may play a role in maintaining the correct localization, protein abundance, and stability of OsGASR9, which is necessary for OsGASR9 to perform its normal function. Studies have shown that HSP genes such as *HSP101* and *HSP60* are suppressed by the overexpression of *GASA5* when plants are exposed to heat stress, suggesting that the *AtGASA5* gene is indirectly involved in the accumulation of HSPs and plays a negative regulatory role in heat tolerance [23]. Whether the interaction between the OsNAL11 and OsGASR9 proteins affects each other’s normal function, how the *OsNAL11* and *OsGASR9* genes affect the expression of genes related to the GA pathway, and which gene is upstream, and which is downstream in the genetic relationship needs to be further investigated.

## 4. Materials and Methods

### 4.1. Experimental Materials and Processing

The test materials in this study were the japonica variety Zhonghua 11 (ZH11), which is used as the wild type (WT), *OsNAL11* (*LOC_Os07g09450*) and *OsGASR9* (*LOC_Os07g40240*) knockout mutants *nal11* (*nal11-1*, *nal11-2*) and *gasr9* (*gasr9-1*, *gasr9-2*) derived from ZH11 using CRISPR/Cas9 gene editing technology (Figure S3). The wild type and two mutant materials used in this experiment were provided by our laboratory. All materials were planted during normal seasons in the Teaching and Research Experimental Field of South China Agricultural University (Guangzhou, China).

For both the wild type and the two mutants, rice seeds were selected at the mature stage 30 days after flowering and dried at 42 °C for 5 days to break seed dormancy. Full-grain seeds were selected as test materials. To test the germination of ZH11 and the *nal11-1*, *nal11-2*, *gasr9-1*, and *gasr9-2* mutants at room temperature, 50 whole and dry seeds were evenly distributed in a 9 cm diameter petri dish with two layers of filter paper, respectively, then added to 10 mL of sterile H_2_O and placed in an incubator at 28 ± 1 °C. To test germination at low temperatures, each sample was supplemented with 10 mL of sterile H_2_O and then placed in an incubator at 15 ± 1 °C with a daily cycle of 12 h of light and 12 h of dark. The standard for germination was that the length of the seed germ reached 1.0 mm. The number of germinated seeds was counted every half day at room temperature and daily at a low temperature, and germination rates and other indicators were calculated accordingly. 30 dry seeds per replicate of ZH11 and the *nal11-1*, *nal11-2*, *gasr9-1*, and *gasr9-2* mutants were sown uniformly in 1 cm-deep soil in a small pot, respectively, placed at 18 ± 1 °C and moistened to simulate direct seeding experiments. Seedling shoot height, seedling fresh weight, and root length were determined after 14 days. Each treatment was replicated three times.

### 4.2. Measurement of Physiological Indices

At 0, 3, 6, and 9 d during low-temperature germination, ten seeds from a single material of the WT or mutants were selected, stored at -80 °C, and then ground in liquid nitrogen. Seven physiological and biochemical parameters, including α-Amylase (α-AMS) activity, malonaldehyde (MDA) content, H_2_O_2_ content, peroxidase (POD) activity, total superoxide dismutase (SOD) activity, catalase (CAT) activity, and anti-superoxide anion activity were then determined. The levels of MDA and H_2_O_2_ were determined using the MDA assay kit A003-1-1 (Malondialdehyde assay kit, Nanjing, China) and the H_2_O_2_ assay kit A064-1-1 (Hydrogen Peroxide assay kit, Nanjing, China), respectively. The activities of α-AMS, CAT, POD, and SOD were detected by α-AMS assay kit C016-1-1 (α-Amylase Assay Kit, Nanjing, China), CAT assay kit A007-1-1 (Catalase assay kit, Nanjing, China), POD assay kit A084-3 (Peroxidase assay kit, Nanjing, China) and SOD assay kit A001-1 (Total Superoxide Dismutase assay kit, Nanjing, China), respectively [50]. The ability of seeds to eliminate superoxide anions was determined using a commercially available inhibition and generation of superoxide anion kit A052-1-1 (Inhibition and produce superoxide anion assay kit, Nanjing, China) [51]. All the kits were supplied by Nanjing Jiancheng Bioengineering Institute, and all the operations were performed according to the manufacturer’s instructions. All data were collected from three replicates. All the seeds described in this study are mature, full, and undamaged after strict selection.

### 4.3. Gene Expression Analysis

Total RNA was extracted from the seeds at 0, 3, 6, and 9 d during seed germination at low temperatures according to the instructions on Ultramicro RNA Rapid Extraction Kit (RN5601, Beijing Aidelai Biotechnology Co., Ltd., Beijing, China). cDNA was reverse transcribed using an Evo M-MLV RT Kit with gDNA Clean for qPCR II (Agbio, Changsha, China) and used for subsequent quantitative analysis of target genes. The quantitative real-time polymerase chain reaction (qRT-PCR) was performed using the AceQ qPCR SYBR Green Master Mix (High ROX Premixed) kit (Vazyme, Nanjing, China). The qRT-PCR reaction system included 10 μL SYBR Green, 3 μL cDNA, and 0.4 μL upstream and downstream primers. The qRT-PCR reaction procedure included 95 °C for 5 min, 95 °C for 10 s and 60 °C for 30 s for 40 cycles, and extension at 72 °C for 3 min. The rice *β-actin* gene (*LOC_Os03g50885*) was used as an internal reference, and the data were sorted by the ΔΔCT method. Expression of the target gene was analyzed with four replicates. All qRT-PCR primers used in this study were designed on the NCBI website (www.ncbi.nlm.nih.gov, accessed on 7 September 2022). The primers used in this study are listed in Supplementary Table S1.

### 4.4. Yeast Two-Hybrid (Y2H) Assays

The Y2H assays were used with the Matchmaker GAL4 two-hybrid system (Clontech, California City, CA, USA) to identify the proteins interacting with OsNAL11. The CDS (Coding sequence) of *OsNAL11* was inserted into the *pGBDT7* vector as bait, while the CDS of *OsGASR9* was inserted into the *pGADT7* vector as prey. The bait and prey plasmids were co-transformed into Y2H Gold yeast cells according to a lithium acetate method. Yeast cells were cultured on a selective medium lacking leucine, tryptophan, histidine, and adenine (SD/−Leu/−Trp/−His/−Ade) according to standard protocols (Clontech, CA, USA) to investigate possible protein–protein interactions. The primers used in this experiment are listed in Supplementary Table S1.

### 4.5. Luciferase (LUC) Complementation Imaging Assays

For the LUC assays, the *pCAMBIA1300-LUC* vector was first linearized with *Kpn I* and *Sal I*. Then, the CDSs of *OsGASR9* and *OsNAL11* amplified by PCR were cloned into the *pCAMBIA1300-nLUC* and *pCAMBIA1300-cLUC* vectors to generate GASR9-nLUC and NAL11-cLUC, respectively. These two fusional expression vectors were transformed into *Agrobacterium* strain GV3101 by heat shock, respectively, and subsequently infiltrated together into the leaf epidermal cells of 3-week-old *N. benthamiana.* After 48 h of incubation, the LUC signals were captured on the transformed leaves using the Night SHADE LB 985 (Berthold Technologies, Bad Wildbad, Germany). The primers used in this experiment are listed in Supplemental Table S1.

### 4.6. Co-Immunoprecipitation (Co-IP) Assays

The CDS fragment of *OsNAL11* was cloned into the *pRTVcHA* vector, which was first linearized with *Bam HI* and *Sac I*. After identification of the resulting construct by Sanger sequencing, the *pRTVcHA-NAL11* fusion vector was obtained. Similarly, the CDS fragment of *OsGASR9* was cloned into the *pRTVcFlag* vector linearized with *Bam HI* and *Hind III* first, and then the *pRTVcFlag-GASR9* fusion expression vector was obtained after Sanger sequencing. The *pRTVcHA-NAL11* and *pRTVcFlag-GASR9* plasmids (test group) were co-transformed into rice protoplasts, with the *pRTVcHA-NAL11* or *pRTVcFlag-GASR9* plasmid alone serving as the control group, respectively. Genetic transformation of rice protoplasts was performed as previously described [52]. After a period of 12–14 h at room temperature, the protoplasts were collected, and the total proteins were extracted. An appropriate amount of Flag magnetic beads was added, and the samples were incubated overnight at 4 °C. The IP products were then eluted and analyzed using Western blot. The target proteins were detected in the IP products of the experimental and the control groups using Flag and HA antibodies, respectively.

### 4.7. Statistical Analysis and Data Plotting

Excel 2016 was used for basic statistics and data analysis, and significant differences among samples were compared using the Student’s *t*-test or analysis of variance (ANOVA) performed with the aid of SPSS (IBM Corp. Released 2019. IBM SPSS Statistics for Windows, Version 26.0. IBM Corp, Armonk, NY, USA). GraphPad Prism 9.5 software (GraphPad Software, Inc., La Jolla, CA, USA) was used for image drawing.

## 5. Conclusions

In summary, at room temperature, the germination rate of *nal11* and *gasr9* mutants is not significantly different from that of the WT, whereas at low temperatures, the germination rate of the mutants is reduced, and the seedlings grow slowly. During germination at low temperatures, there was more accumulation of MDA and ROS in the seeds of *nal11* and *gasr9* mutants. In addition, the active GA content was significantly reduced in the two types of mutants, especially GA4 and GA7. The expression of genes related to the GA pathway was also significantly altered compared to the wild type. This suggests that the genes *OsNAL11* and *OsGASR9* play a specific role in the low-temperature germination of rice seeds. The interaction between the OsNAL11 and OsGASR9 proteins was confirmed by Y2H, LUC, and Co-IP experiments. This shows that they have a common role in the low-temperature germination of rice seeds, possibly involving the ROS and GA pathways.

The molecular mechanism of the role of the proteins OsNAL11 and OsGASR9 in the low-temperature germination of rice seeds is still unclear. OsGASR9 may be a key factor affecting GA levels, and further research is needed to determine how it affects GA synthesis, degradation, and signal transduction, thereby affecting the expression of downstream genes. It is also unknown whether the heat shock protein OsNAL11 can act as a molecular chaperone to regulate the activity of OsGASR9. The significance of the interaction between OsNAL11 and OsGASR9 proteins still needs to be further investigated.

## Data Availability

All of the datasets are included within the article and its additional files.

## Supplementary Materials

The following supporting information can be downloaded at: https://www.mdpi.com/article/10.3390/ijms252011291/s1.
