# Supplementary figures and images for: OsNAL11 and OsGASR9 Regulate the Low-Temperature Germination of Rice Seeds by Affecting GA Content
Source: Int J Mol Sci. 2024 Oct 20;25(20):11291. doi: 10.3390/ijms252011291 (PMC11508740; doi:10.3390/ijms252011291)

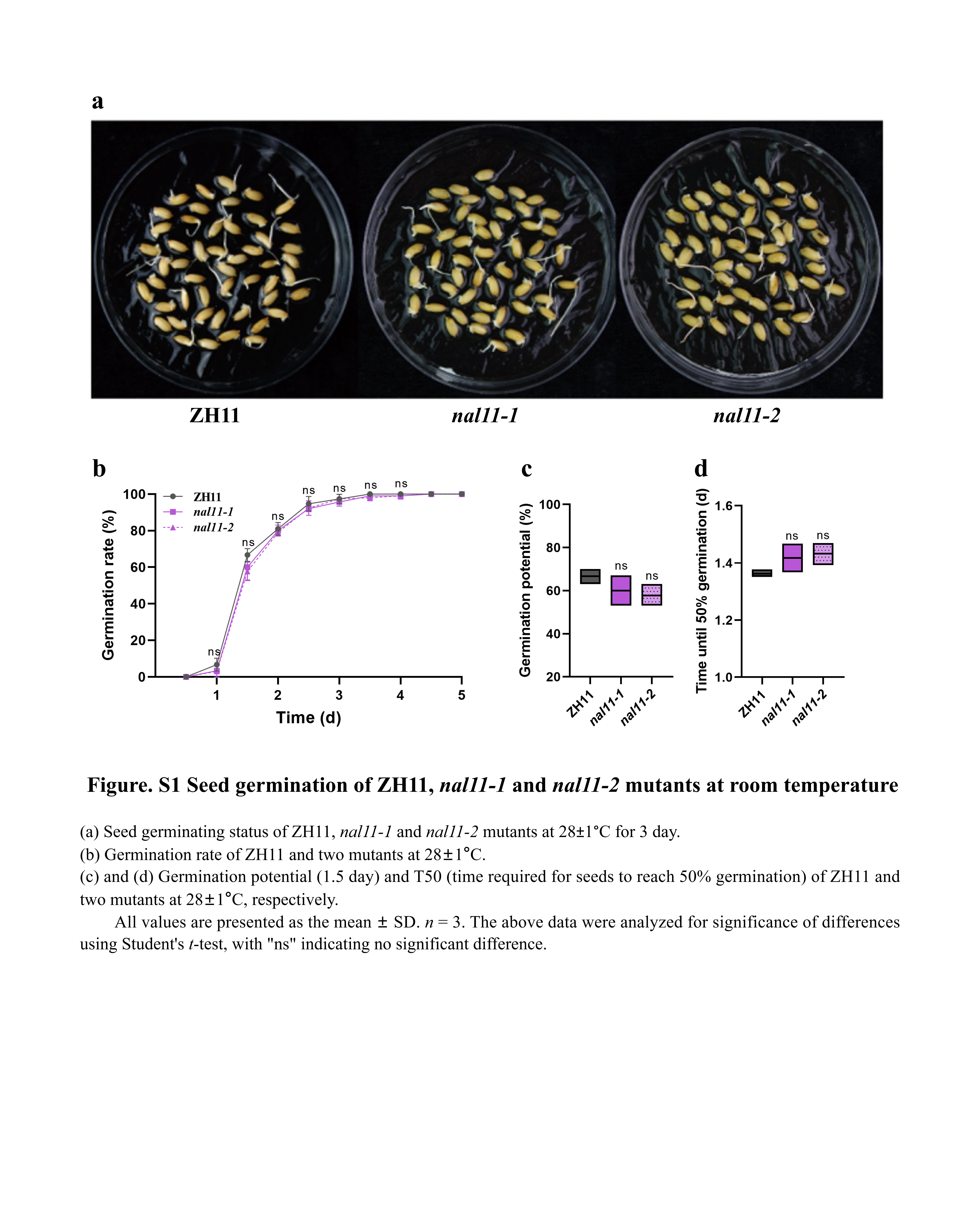

Supplement: Supplementary file 1 [file ijms-25-11291-s001.zip › 20241023-ijms-3239146-supplementary-revised-Supplemental Figure. S1.tif]

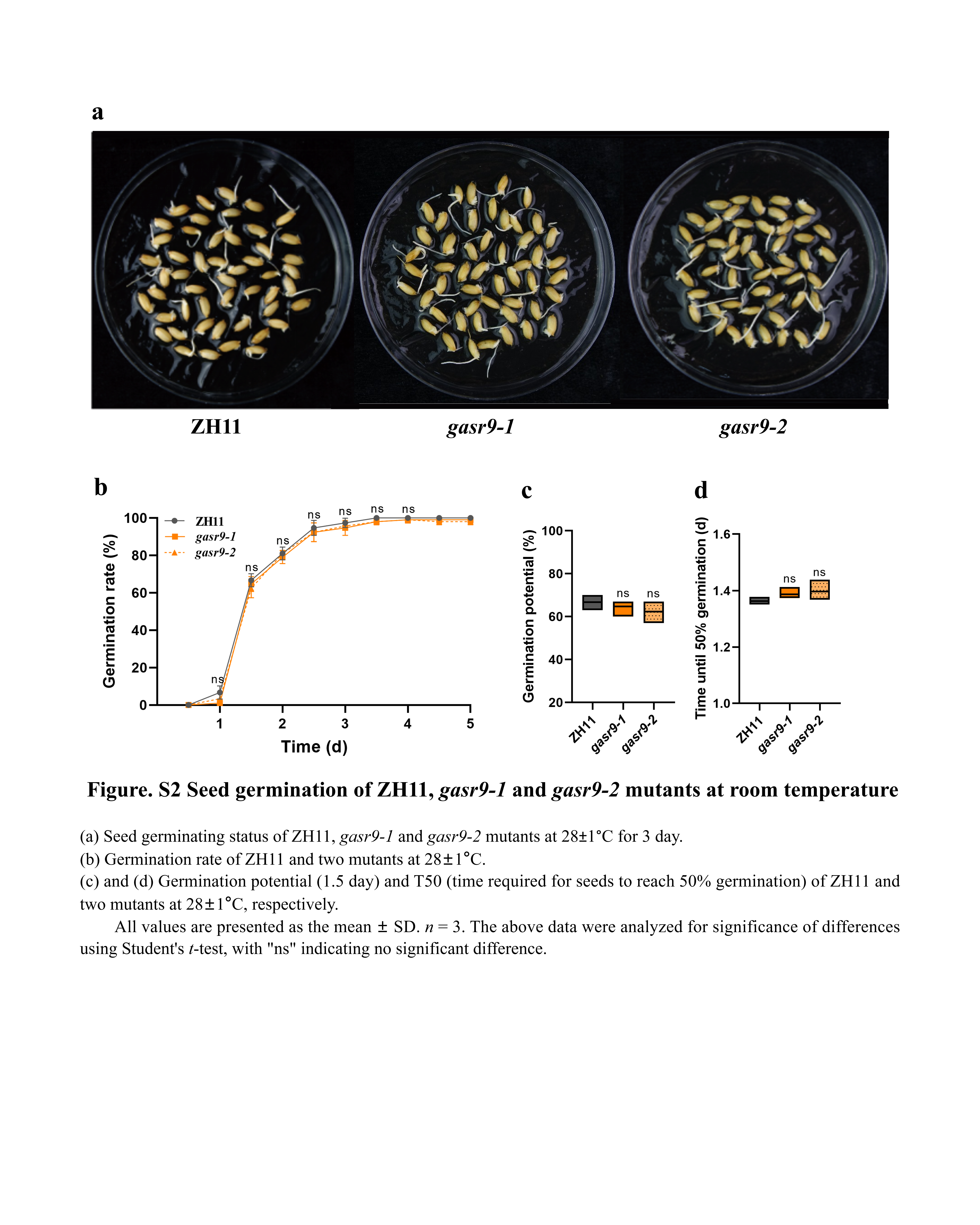

Supplement: Supplementary file 1 [file ijms-25-11291-s001.zip › 20241023-ijms-3239146-supplementary-revised-Supplemental Figure. S2.tif]

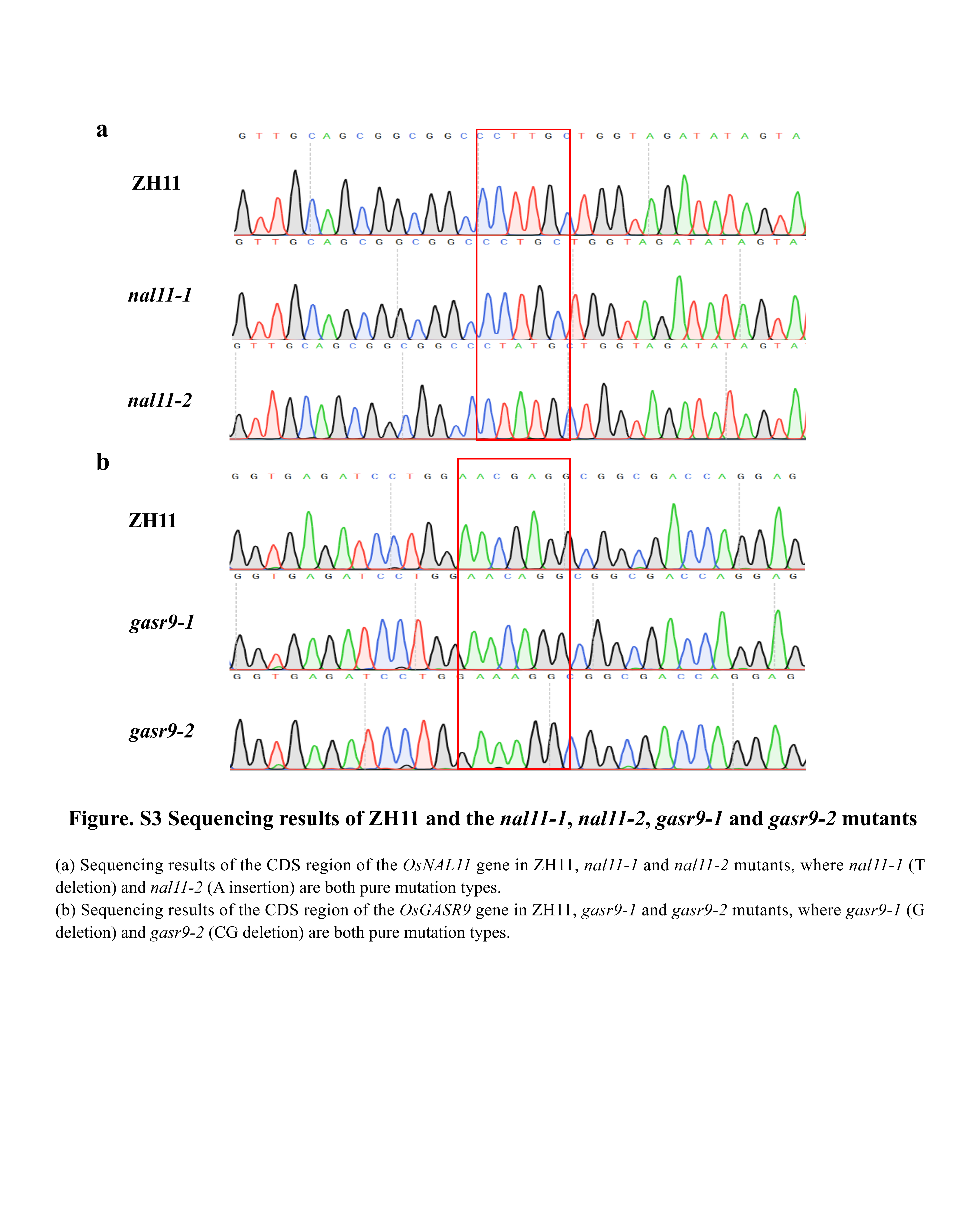

Supplement: Supplementary file 1 [file ijms-25-11291-s001.zip › 20241023-ijms-3239146-supplementary-revised-Supplemental Figure. S3.tif]
